# Association of allostatic load with brain structure and cognitive ability in later life

**DOI:** 10.1016/j.neurobiolaging.2014.12.020

**Published:** 2015-03

**Authors:** Tom Booth, Natalie A. Royle, Janie Corley, Alan J. Gow, Maria del C. Valdés Hernández, Susana Muñoz Maniega, Stuart J. Ritchie, Mark E. Bastin, John M. Starr, Joanna M. Wardlaw, Ian J. Deary

**Affiliations:** a Centre for Cognitive Ageing and Cognitive Epidemiology, The University of Edinburgh, Edinburgh, UK; b Department of Psychology, The University of Edinburgh, Edinburgh, UK; c Brain Research Imaging Centre, Division of Neuroimaging Sciences, The University of Edinburgh, Edinburgh, UK; d Department of Psychology, School of Life Sciences, Heriot-Watt University, Edinburgh, UK; e Geriatric Medicine Unit, The University of Edinburgh, Western General Hospital, Edinburgh, UK

**Keywords:** Allostatic load, Cognitive ability, White-matter volume, Gray-matter volume, Hippocampal volume, Structural equation modeling

## Abstract

Allostatic load (AL) has been proposed as a general framework for understanding the cumulative effects of life stress on individuals. Despite growing interest in AL, limited research has been conducted on aging samples. We consider the association of AL (operationalized by a range of inflammatory, cardiovascular, and metabolic measures) with a range of brain volume measurements and cognitive ability in a large cohort sample of older adults (*n* = 658, mean age = 72.5 years, standard deviation = 0.7) using structural equation modeling. AL was significantly inversely associated with total brain volume (range of standardized β = −0.16 to −0.20) and white-matter volume (−0.35 to −0.36) and positively with hippocampal volume (0.10–0.15) but not gray-matter volume (0.04). AL was also significantly inversely associated with general cognitive ability (range β = −0.13 to −0.20), processing speed (−0.20 to −0.22), and knowledge (−0.18 to −0.20) but not memory or nonverbal reasoning. The associations of AL with cognitive abilities were not mediated by these brain volume measures. AL did not predict cognitive change from age 11 to approximately age 73. The findings suggest a link between AL and later life brain health and cognitive functioning.

## 1 Introduction

The concept of “allostasis” has played a prominent role in recent stress research in both human and nonhuman animals. In brief, individuals are exposed to multiple stressors, both social and environmental, which induce a stress response. Allostasis refers to the process of fluctuating activity of the body's physiological systems in response to such stressors (Sterling and Eyer, 1988). The primary systems of allostasis and the stress response include the neuroendocrine, sympathetic nervous, immune, metabolic, cardiovascular, and hypothalamic-pituitary-adrenal axis (Seplaki et al., 2006). Common markers of allostatic load (AL) in the applied research include blood pressure, pulse pressure, heart rate variability, blood glucose, body mass index (BMI), high- and low-density lipoproteins (LDLs), fibrinogen, C-reactive protein (CRP), interleukin-6 (IL-6), epinephrine, and norepinephrine, to name but a few (see Karlamangla et al., 2013; Juster et al., 2010). Regular or acute exposure to stressors may result in chronic imbalance across 1 or multiple of these systems, referred to as the “allostatic state.” Over time, the biological aftermath of allostatic states accumulates, resulting in AL. AL, therefore, can be thought of as the biological “wear and tear” on the body as a result of its inability to cope with the stressful stimuli and events (McEwen and Stellar, 1993). Two principal concepts in AL theory are important with respect to the present study; namely, cumulative load and the central role played by the brain in allostasis.

As has been noted earlier, AL theoretically represents the accumulated damage of the allostatic process on the body over time. Therefore, time, in the case of the human life course, development and aging are important aspects of research into AL. Indeed, many models of life stress and AL focus on phasic periods of increased sensitivity to the detrimental effects of stressors in development (e.g., Del Giudice et al., 2011, Adaptive Calibration Model), whereas the importance of studying the impact of AL in aging has also been noted (Ganzel et al., 2010; Karlamangla et al., 2002). It is also of interest to note the overlap between suggested lists of biomarkers of aging (e.g., Dowd and Goldman, 2006) and markers of AL (e.g., Juster et al., 2010).

AL is conceptualized as a cumulative process. As such, it is plausible to suggest that even if individuals have low AL during early adulthood, the passage of time may lead to increased AL in later life. Crimmins et al. (2003) found, using the National Health and Nutrition Examination Survey study data, that, whereas AL increased from the 20’s to the 60’s, levels of AL stabilized during the 70’s and 80’s. However, caution is required in interpretation of these trends as they are based on cross-sectional data and, therefore, are likely to partially reflect a survival effect, whereby those lowest in AL reach older ages.

A number of studies have considered the impact of AL on mortality (e.g., Goldman et al., 2006; Gruenewald et al., 2006; Karlamangla et al., 2006; Seeman et al., 2004) and cognitive and health declines in aging (for a summary, see Juster et al., 2010, Table 1). With respect to cognitive ability, a recent cross-sectional study by Karlamangla et al. (2013) using data from a subset of the Midlife in the United States Study (*n* = 1076, mean age = 57 [range, 49–66] years) found that AL significantly negatively predicted episodic memory score (*p* < 0.001) and executive function (*p* < 0.001) accounting for 4.9% and 7.3% of the variance, respectively. These results remained significant after adjusting for covariates. In that study, AL was measured based on 24 biomarkers taking percentage risk cut points to produce a single sum score. In a series of analyses using the McArthur Studies of Successful Aging, Seeman et al. (1997), (2001) showed AL, measured by 10 biological markers, significantly predicted cognitive decline across 3 (*r* = −0.08, *p* < 0.05, specifically here for memory) and 7 (unstandardized *b* = −0.58, *p* = 0.03) years. In both studies, cognitive ability was assessed by a sum score of tasks designed to measure language, abstraction, spatial ability, and memory, with change assessed by including baseline ability as a covariate.

In a similar series of studies on the Taiwanese Social Environment and Biomarkers of Aging sample, Seplaki et al. (2006) investigated AL associations with cognitive ability and using multiple different quantitative methods. For example, Seplaki et al. (2006) compared the predictive power of a simple risk sum score of AL to a grade of membership model score for AL that captures an individual's difference from a “low-risk” AL profile based on AL biomarkers and a cognitive ability score measured by modified versions of the Rey Auditory Verbal Learning Test and the Digit Symbol Backward Test. They found that both the sum score and the grade of membership score significantly and negatively predicted cognitive ability (*p <* 0.01 and *p <* 0.05, respectively). Although not intended to be exhaustive, these studies highlight the accumulating evidence that greater AL is associated with lower level and decline in cognitive ability in aging.

Second, central to theorizing on allostasis and AL has been the role of the brain as the central mediator of the stress response (for recent discussions, see Ganzel and Morris, 2011; Ganzel et al., 2010; McEwen and Gianaros, 2010). In particular, the hippocampus, amygdala, hypothalamus, and prefrontal cortex have all been proposed as primary locations for both the adaptive and maladaptive effects of stress responsivity (McEwen and Gianaros, 2010). Much research has been published exploring the associations among negative life experiences, stress and related neurochemical exposures, and brain integrity in both younger and older samples. For example, Ansell et al. (2012) showed that a measure of cumulative adversity, the cumulative adversity interview, which assesses stressful life events and perceived stressors, was associated with reduced gray-matter volumes in a number of brain regions. Satizabal et al. (2012) found significant associations between IL-6 and CRP (that are regularly used as biomarkers of AL) with brain white-matter hyperintensities and total gray-matter volume and IL-6 with hippocampal volumes in a sample of 1841 participants aged 65–80 (mean 72.5) years. Furthermore, as has been noted by Ganzel et al. (2010), there is an overlap between those regions of the brain known to be affected by stress and those that undergo greatest atrophy in aging.

However, despite the central role of the brain in allostasis, the growing body of research on the impact of AL on cognitive ability in aging and the well-known associations between brain integrity and cognitive ability across the life course (for reviews, see Deary et al., 2010a; Salthouse, 2011), there remains little to no research exploring the interplay among AL, cognitive functioning, and brain structure in aging. As such, the primary aim of this study was to report on such associations in a large generally healthy cohort of community-dwelling older adults. Specifically, we test whether AL is associated with general cognitive ability, processing speed, and a range of brain volume measures in older age. Further, we test whether brain volume mediates any association of AL with cognitive ability in later life.

## 2 Methods

### 2.1 Participants

Participants were drawn from the Lothian Birth Cohort 1936 (LBC1936). The LBC1936 is a longitudinal study of aging comprising surviving members of the Scottish Mental Survey 1947, who were resident in the Lothians (the City of Edinburgh and its surrounding area) at the time of the recruitment. At wave 1 recruitment, 1091 (male = 548 and female = 543) generally healthy participants with a mean age of 69.5 (standard deviation [SD] = 0.8) years entered the study. At wave 2, 866 (male = 448 and female = 418) participants returned with a mean age of 72.5 (SD = 0.7) years. Further details can be found in the study protocol papers (see Deary et al., 2007, 2011). Of the 866, 700 underwent brain magnetic resonance imaging (MRI), of whom 679 had usable imaging measures for the present study (Wardlaw et al., 2011). A further 21 participants were removed who did not have blood examination data. Last, 25 participants were removed who scored lower than 26 on the Mini-Mental State Examination (Folstein et al., 1975). Scores less than 26 have been argued to be indicative of mild cognitive impairment and which may be indicative of early signs of dementia. This resulted in a final sample size of 633. With the exception of age 11 cognitive ability and the socioeconomic variables described subsequently, all measures used in the present study were taken from wave 2 of testing.

### 2.2 Measures

#### 2.2.1 Allostatic load

The present study is based on 10 biomarkers identified to represent different contributing factors to AL, namely, fibrinogen, triglyceride, high-density lipoprotein (HDL) and LDL, from which total cholesterol and cholesterol-HDL ratio were obtained, glycated hemoglobin, CRP, IL-6, BMI, and mean systolic blood pressure (SBP) and diastolic blood pressure (DBP). Blood samples were taken during participants' physical examination at wave 2 of testing. Information on the collection of these variables has been reported previously (Booth et al., 2013a) and is reproduced here in Supplementary Material E1.

To the best of the authors' knowledge, all participants were well at the time of testing, but we cannot rule out imminent or recent influenza, sinusitis, or other potential short-term causes of raised inflammatory markers.

#### 2.2.2 Cognitive ability

The suite of the cognitive tests was designed to measure multiple aspects of cognitive ability known to be important in aging. They included the Block Design, Matrix Reasoning, Digit Symbol Coding, Symbol Search, and Letter-Number Sequencing subtests of the Wechsler Adult Intelligence Scale, Logical Memory (immediate and delayed recall), Verbal Paired Associates (immediate and delayed recall), Digit Span (backward), Spatial Span (forward and backward) subtests of the Wechsler Memory Scale; the National Adult Reading Test; the Wechsler Test of Adult Reading; verbal fluency; simple and 4-choice reaction time tasks; and finally an inspection time task of visual processing efficiency. To test whether contemporaneous associations between AL and cognitive ability hold after controlling for prior ability, we also included participants' cognitive ability scores on the Moray House Test No. 12 from the Scottish Mental Survey 1947, completed when participants were aged 11 years. Specific details about each test can be found in the LBC1936 study protocol (Deary et al., 2007) and in the Supplementary Material E2.

#### 2.2.3 Image acquisition

Structural MRI data were obtained from a GE Signa Horizon HDxt 1.5-T clinical scanner (General Electric, Milwaukee, WI, USA) using a self-shielding gradient set with maximum gradient strength of 33 m/Tm and an 8-channel phased-array head coil. The examination included T2-weighted (T2W) (repetition time [TR]/echo time [TE] = 11,320/102 ms), T2*W (TR/TE = 940/15 ms), and fluid-attenuated inversion recovery–weighted (TR/TE/inversion time = 9000/140/2200 ms) axial scans and a high-resolution T1W volume sequence (TR/TE/inversion time = 9.8/4/500 ms) acquired in the coronal plane. The full procedure (which also included diffusion-tensor–MRI measurements not used for the present study) took approximately 70 minutes for each participant. Wardlaw et al. (2011) provide all details of the imaging protocol.

#### 2.2.4 Image analyses

All image analyses were performed by trained analysts blinded to the participant information (see supplementary Material E3). Intracranial volume (ICV) was defined as including the contents within the inner skull table with its inferior limit in the axial slice just superior to the tip of the odontoid peg at the foramen magnum and superior to the inferior limits of the cerebellar tonsils (Wardlaw et al., 2011). The ICV, which includes brain tissue, cerebrospinal fluid (CSF), veins, and dura, was obtained semiautomatically using the T2*W sequence, with the Object Extraction Tool in Analyze 9.0 (AnalyzeDirect Inc, Mayo Clinic) providing an initial segmentation that was manually edited to remove erroneous structures.

CSF and white and gray matter were extracted using MCMxxxVI (Valdés Hernández et al., 2010, 2012). The combination of T2*W and fluid-attenuated inversion recovery volumes were used to extract CSF, and the CSF masks were then subtracted from the ICV to provide a measure of total brain volume. White-matter masks were produced by fusing T2W and T1W volumes to give good white- and/or gray-matter contrast; white-matter hyperintensities were not included in the white-matter segmentation. Gray-matter masks were calculated by subtracting the white-matter masks and white-matter hyperintensity binary masks from the brain tissue masks created previously.

Hippocampal volumes were acquired from the T1W scans using the freely available software FSL_FIRST (Patenaude et al., 2011); and all outputs were visually inspected and manually edited, where necessary, by a trained image analyst. All volumetric measures were adjusted for ICV before use in the structural models.

#### 2.2.5 Socioeconomic variables

At testing wave 1, participants provided the following 3 variables at an interview. First, they noted their number of years of formal, full-time education. Second, they provided the socioeconomic classification of the occupation held by their father at the time of their birth in 1936. This was rated on the scale of the General Register Office's Census 1951 *Classification of Occupations*, which rates occupations on a 5-class scale from the professional (class I) to unskilled (class V). Third, they provided the socioeconomic classification of the most prestigious job they held before retirement, rated on the same scale as the father's occupation, with one difference: class III was split into manual and nonmanual occupations (Office of Population Censuses and Surveys, 1951, 1980).

### 2.3 Analysis strategy

We applied structural equation modeling (SEM) first to establish plausible measurement models for the constructs of interest (AL and cognitive ability) and to test the proposed associations between the constructs (see Section 2.3.2). SEM has a number of advantages over other statistical approaches, and it specifically (i) allows for the direct modeling of error-free latent constructs of interest and their associations with other variables in the model, (ii) can handle multiple data types and non-normality in indicator variables, (iii) can handle missing data, (iv) is a multivariate approach, and (v) provides a large number of diagnostic indices for evaluation of the adequacy of the model. Supplementary Material E4 provides a brief introduction to SEM.

#### 2.3.1 Measurement models

For both AL and cognitive abilities, we fit confirmatory bifactor measurement models based on the previous studies on the current sample (Booth et al., 2013a, 2013b). Booth et al. (2013a) discuss the benefits of the bifactor model for neuroimaging studies of cognitive ability (for general discussion, see also Colom and Thompson, 2011; Reise, 2012). Briefly, the bifactor model characterizes the observed correlations among variables as being accounted for by a single general factor and specific factors related to smaller clusters of variables. As such, the bifactor model partitions variance in the observed variables into that which is common to all variables and that which is common to smaller subsets of variables (Reise, 2012). Importantly, this provides robust estimates for the general latent factor and estimates of specific factors that are free of variance associated with the general factor. Thus, bifactor models are particularly useful when researchers are interested in estimates of a general factor, here general cognitive ability (*g*) and AL, and independent latent estimates of specific factors, here specific abilities.

For cognitive abilities, we reestimate the model presented in Booth et al. (2013a) in the current sample. Briefly, 5 latent factors were estimated: *g* (loaded by all 18 subtests); processing speed (loaded by Digit Symbol, simple reaction time, choice reaction time, and Symbol Search and inspection time); verbal memory (loaded by Logical Memory and Verbal Paired Associates [immediate and delayed recall]); nonverbal reasoning (loaded by Matrix Reasoning, Block Design, Digit Span [backward], Letter-Number Sequencing and Spatial Span [forward and backward]); and knowledge (loaded by National Adult Reading Test, Wechsler Test of Adult Reading, and verbal fluency). All factors were uncorrelated, meaning the estimates of each construct were independent. Latent factors were identified by fixing the variance of the latent factor to 1.0 (Bollen, 1989).

AL has been previously assessed in the current sample using higher order SEMs (Booth et al., 2013a). We use the same set of indicator variables but apply confirmatory bifactor modeling. Four latent factors were estimated: AL (loaded by all 10 indicators), blood pressure (loaded by mean DBP and SBP), metabolism (loaded by triglyceride, HDL, LDL, glycated hemoglobin, and BMI), and inflammation (loaded by fibrinogen, CRP, and IL-6). As with the cognitive model, latent variables were identified by fixing their variance to 1.0, and the latent variables were uncorrelated (independent).

Booth et al. (2013a) previously found that the measurement of AL varied across groups dependent on their medication status. Therefore, medications were classified (by JMS) into anti-inflammatory, antihypertensive, lipid lowering, insulin, and other diabetes medications, with medication status defined as taking any ≥1 of these medications and tested both the AL and cognitive ability measurement models for equivalence across medication status groups.

We use multigroup SEMs to first test for measurement invariance of the latent variables across groups. Tests of measurement invariance provide a formal test of whether the latent constructs of interest can be considered equivalent across groups (Millsap, 2011). If measurement invariance is established, structural parameters (regression paths among latent variables) can be meaningfully compared across groups. In multigroup SEM, this is achieved by testing the decrease in model fit when parameters are constrained to equality. Collectively, the series of multigroup models provides a rigorous statistical assessment of whether a whole-sample analysis is meaningful (for details, see Supplementary Material E4).

#### 2.3.2 Structural models

**Fig. 1:**
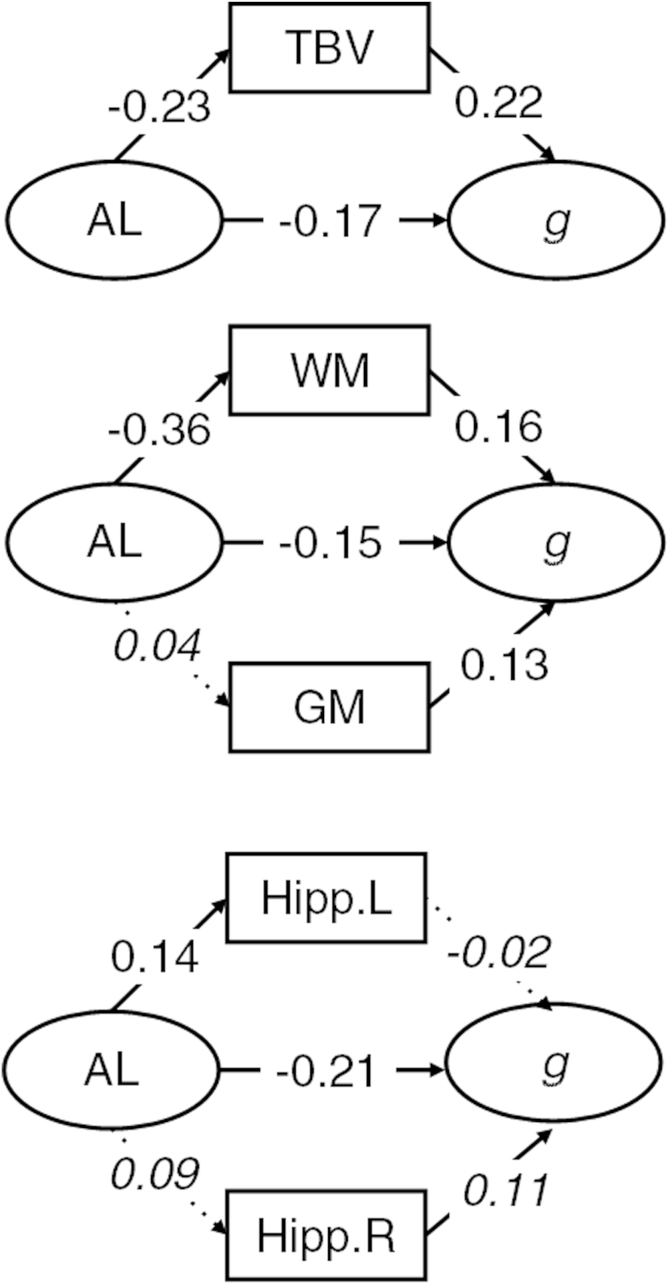
Structural model with standardized parameter estimates for general cognitive ability. Ellipses indicate latent variables where measurement model parameters are excluded for clarity of presentation. Rectangles indicate observed variables. Italicized values with dotted paths are nonsignificant. Nonitalicized values are significant at *p* < 0.05. Abbreviations: AL, allostatic load; *g*, general cognitive ability; GM, gray-matter volume; Hipp. L, left-hemisphere hippocampal volume; Hipp. R, right-hemisphere hippocampal volume; TBV, total brain volume; WM, white-matter volume.

**Fig. 2:**
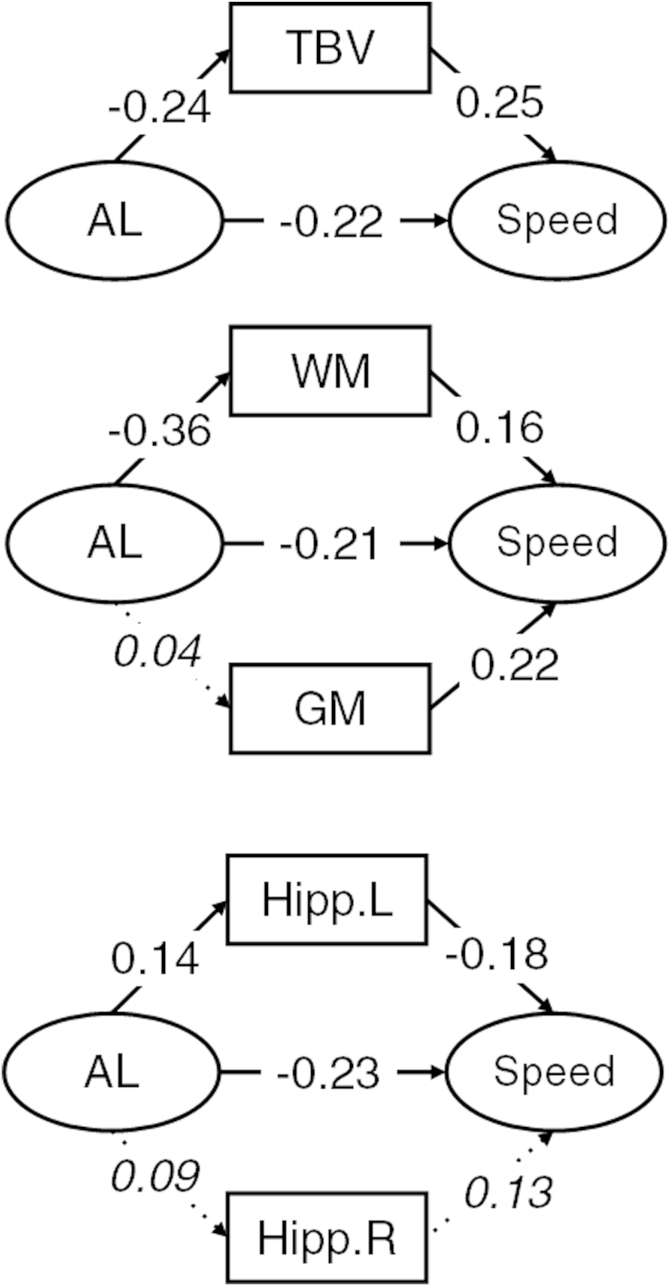
Structural model with standardized parameter estimates for processing speed. Ellipses indicate latent variables where measurement model parameters are excluded for clarity of presentation. Rectangles indicate observed variables. Italicized values with dotted paths are nonsignificant. Nonitalicized values are significant at *p* < 0.05. Abbreviations: AL, allostatic load; GM, gray-matter volume; Hipp. L, left-hemisphere hippocampal volume; Hipp. R, right-hemisphere hippocampal volume; speed, processing speed; TBV, total brain volume; WM, white-matter volume.

**Fig. 3:**
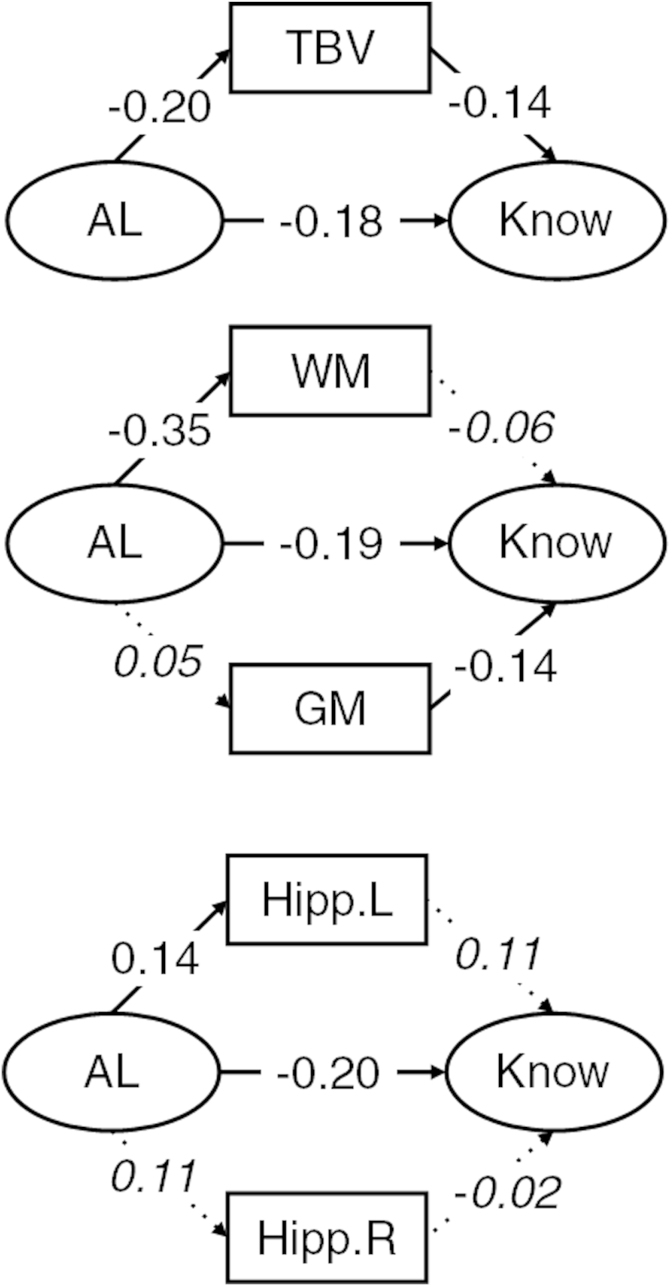
Structural model with standardized parameter estimates for knowledge. Ellipses indicate latent variables where measurement model parameters are excluded for clarity of presentation. Rectangles indicate observed variables. Italicized values with dotted paths are nonsignificant. Nonitalicized values are significant at *p* < 0.05. Abbreviations: AL, allostatic load; GM, gray-matter volume; Hipp. L, left-hemisphere hippocampal volume; Hipp. R, right-hemisphere hippocampal volume; know, knowledge; TBV, total brain volume; WM, white-matter volume.

A series of models were then tested in which the associations among AL, cognitive abilities, and brain imaging variables were estimated. Across all models, we considered whether the direct association of AL with cognitive abilities was attenuated by inclusion of the brain imaging variables as mediators. We also considered the direct associations of AL on total brain, gray- and white-matter volumes, and hippocampal volumes (see Figs. 1–3).

#### 2.3.3 Model testing

All models were estimated in Mplus 6.0 (Muthen and Muthen, 2010) using robust maximum-likelihood estimation as a number of variables had small degrees of skew and kurtosis. Small amounts of missing data were present (see Table 1, minimum covariance coverage = 92%). As such, full-information maximum-likelihood estimation was used, as this is considered to be one of the most robust methods for dealing with the missing data (Enders, 2010).

Model fit was assessed based on the comparative fit index (CFI), Tucker-Lewis index (TLI), root-mean-square error of approximation (RMSEA), and standardized root-mean residual (SRMR). Based on the extant literature, investigating the performance of model fit indices (e.g., Schermelleh-Engel et al., 2003), we consider model fit values >0.95 for the CFI and TLI, <0.05 for the RMSEA, and <0.06 for the SRMR to be indicative of good model fit. We note, however, that all cutoffs are to some degree arbitrary (Marsh et al., 2005) but consider these values to provide reasonable assurance that models are adequate representations of the data and that parameters can be substantively interpreted. Input variables for all analyses were standardized residuals after regressing on age and sex for the cognitive variables and age, sex, handedness, and ICV for the volumetric imaging variables. We controlled for ICV to take account of differences in head size across participants. Controlling for age may be important in understanding whether the effects of AL in later life are independent of chronological age.

## 3 Results

Descriptive statistics for all variables are shown in Table 1. Both CRP and IL-6 had large skew and kurtosis. As such, and in keeping with the previous studies, we log transformed these variables. As can be seen from Table 1, log transformation was successful in reducing the skew and kurtosis of CRP and IL-6.

The results of the multigroup measurement models suggested that both the AL model and the cognitive ability model were invariant across medication status. These findings differ from those in Booth et al. (2013a), (2013b). These differences are driven by the difference in model specification (for further details, see Supplementary Material E5).

### 3.1 Measurement models

The measurement model for cognitive ability showed good fit to the data (χ^2^ = 313.02 [114], *p* < 0.001; CFI = 0.958; TLI = 0.944; RMSEA = 0.053, 95% confidence interval = 0.046–0.060; and SRMR = 0.045). The structural diagram is shown in Supplementary Material E6. The absolute magnitude of the factor loadings for *g* were all significant and ranged between 0.23 and 0.64, suggesting the general ability factor accounted for between 5.3% and 41.0% of variance in the subtests.

The measurement model for AL also showed good fit to the data (χ^2^ = 68.34 [26], *p* < 0.001; CFI = 0.956; TLI = 0.923; RMSEA = 0.051, 95% confidence interval = 0.036–0.066; and SRMR = 0.036). The structural diagram is shown in Supplementary Material E6. The factor loadings for the AL latent factor were variable. The loadings for fibrinogen and SBP and DBP were not significant. The absolute magnitude of loadings for the remaining biomarkers ranged from 0.30 to 0.57 suggesting between 9.0% and 32.5% of variance is explained by AL. As such, AL in the present study is primarily defined by inflammatory and metabolism biomarkers.

### 3.2 Bivariate correlations

Before conducting the structural analyses, we computed the simple correlations between the main constructs of interest (Table 2). AL had small but significant negative associations with total brain volume, white-matter volume, and bilaterally positively with hippocampal volume and is also significantly associated with *g*, processing speed and knowledge. Age 11 IQ showed a strong association with *g* but was also significantly associated with knowledge (*r* = 0.40), and to a lesser extent AL (*r* = −0.11).

### 3.3 Structural models

All structural models (with and without the inclusion of brain imaging variables) showed good fit to the data (range CFI = 0.939–0.952; range TLI = 0.934–0.942; range RMSEA = 0.036–0.041; and range SRMR = 0.044–0.056). We first estimated the models without brain variables. In these models, the direct associations of AL with *g* (standardized β = −0.20, *p <* 0.01), processing speed (β = −0.25, *p<* 0.01), and knowledge (β = −0.20, *p <* 0.03) were all significant. The direct associations of AL with verbal declarative memory and nonverbal reasoning were all nonsignificant.

Next, we estimated the structural models including the brain imaging variables. Estimates of the structural parameters for the full structural models involving *g*, processing speed, and knowledge are shown in Figs. 1–3, respectively. AL showed a significant association (Figs. 1–3) with total brain volume (range β = −0.20 to −0.24, all *p* values *<* 0.02). There was a significant association of AL with white-matter volume (range β = −0.35 to −0.36, *p* values *<* 0.01) but not gray matter (all *p* values > 0.30). AL was associated significantly with left hippocampal volume (β = 0.14 in all models, *p* values < 0.02) but not right hippocampal volume (all *p* values *>* 0.05).

Total brain (Fig. 1), white- and gray-matter (Fig. 2), and hippocampal (Fig. 3) volumes did not significantly attenuate the association between AL and *g*, processing speed, or knowledge.

Last, we considered the degree of attenuation of the direct effect of AL on the 3 cognitive ability measures. We compare the magnitude of the direct effect from the simple models including only AL and cognitive variables (discussed earlier) to those that include brain imaging measures as potential mediators. For *g* and processing speed, the largest attenuations (Δ = 0.05 and 0.04, respectively) were in models including gray- and white-matter volumes, whereas in the models for knowledge, the direct association of AL remained largely unchanged in all models (maximum Δ = 0.02 in the total brain volume model).

### 3.4 Controlling for childhood cognitive ability

Next, we considered whether the associations of AL and brain imaging measures with cognitive abilities remained significant after controlling for childhood IQ. Prior ability is known to be the strongest predictor of current ability and, as such, is an important covariate. Furthermore, inclusion of prior ability allows us to consider if AL is associated with change in cognitive ability. Specifically, the residual of a given variable, here cognitive ability, after regressing out variance associated with this variable from an earlier point in time, represents the deviation from the mean of the previous time point or the change over time. Inclusion of age 11 IQ score as a predictor of AL and later life cognitive ability attenuated the associations between AL and *g* (attenuation range Δ = −0.06 to −0.11 standardized units, all now not significant) and knowledge (range Δ = −0.09 to −0.11, all now not significant) and resulted in nonsignificant direct paths. However, age 11 IQ did not attenuate the association between AL and processing speed (largest attenuation Δ = −0.02); in all models, the AL-speed association remained significant.

### 3.5 Controlling for socioeconomic variables

Previous results indicate that higher socioeconomic status (SES) may be protective against AL (e.g., Evans and Kim, 2007; Seeman et al., 2010). Bivariate correlations (Table 1, lower 3 rows) indicated that the 3 socioeconomic measures used, years of education, childhood SES, and adulthood SES, had some significant relations to AL and to the cognitive variables. For these reasons, we reran our series of models, this time controlling the latent AL variable and the later-life cognitive ability factor for, first, education, and, second, both the participant's SES from childhood and from adulthood. In the models controlling for education, the relation of AL to *g* and to knowledge was attenuated to nonsignificance in all but one model, which was the model including *g* and the hippocampus (range Δ = 0.07–0.10 standardized units across all 6 models). As in the models controlling for age 11 IQ, education had no effect on the significance of the relation between AL and speed, with a mean Δ = 0.01 across the 3 models.

In the models controlling for both the SES variables, the magnitude of the relation of AL to *g* was still significant in the models including total brain volume and the hippocampus but not in the model including white and gray matter (range Δ = 0.01–0.08 across the 3 models). The magnitude of the relation of AL to knowledge and to speed was reduced to nonsignificance for all models (range Δ = 0.01–0.07 across the 6 models). However, the average magnitude of the attenuation was similar for all models (mean Δ = 0.04 for *g*, 0.05 for knowledge, and 0.06 for speed).

## 4 Discussion

The current findings suggest that AL is associated with a range of brain volume measurements in later life, with the strongest effect seen with white-matter volume. AL is also associated with a number of dimensions of cognitive ability important in cognitive aging, specifically general cognitive ability (*g*), processing speed, and knowledge. AL at age 73 was associated with IQ scores at age 11. Although these brain volumes are also associated with later-life cognitive ability, they do not mediate the association between AL and cognitive ability, suggesting largely independent causes for the associations between AL and cognitive and brain variables. Finally, after controlling for childhood cognitive ability, the associations between AL and *g* and knowledge became nonsignificant, suggesting that AL was not associated with lifetime cognitive change. The association between AL and processing speed remained significant.

The estimates of the associations between AL and various cognitive abilities relevant to aging (*g*, processing speed and knowledge) suggest that as AL increases, individuals' scores on a wide variety of cognitive tasks decrease. Importantly, the associations of AL with processing speed and knowledge are independent of *g* because of the application of the bifactor model. The use of the bifactor model is a major strength of the present study. The ability to separate variance because of *g* from variance because of specific cognitive abilities has been argued to be crucial to our understanding of the neuroanatomic underpinnings of cognitive functioning (Booth et al., 2013a, 2013b; Colom and Thompson, 2011).

When we controlled for prior cognitive ability (age 11 IQ score) or educational duration, the associations of AL with both *g* and knowledge mostly became nonsignificant. That is, AL was not associated with these measures of cognitive change across the life course. Given that a small (although nonsignificant) effect remains, the lack of significance after controlling for prior ability or education may reflect a lack of statistical power to detect an association in the presence of limited cognitive change across the lifespan for the current sample (see Deary et al., 2013). On the other hand, given that the prior cognitive ability and education measures reflect variables from earlier in life, these results may imply that low childhood cognitive ability or poor educational attainment leads to higher AL (perhaps via poorer decision-making ability or lower performance in educational and occupational situations leading to a more stressful life course), which is an explanation that is in agreement with other findings in the field of cognitive epidemiology (Deary et al., 2010b), in addition to being associated with later-life ability. Such a “common-cause” account (Deary, 2012) would imply that AL had no causal effect on later-life cognitive ability. Our study was not able to tease apart these different causal interpretations. Future research, perhaps in samples with repeated longitudinal measures of AL beginning early in life, could come closer to understanding the causal direction of the relation, indeed, possibly the dynamic reciprocal association, between intelligence and AL through the life course.

The association between AL and processing speed remained significant after control for age 11 IQ, although it is important to note that the age 11 IQ measure (Moray House Test) does not assess processing speed after *g* has been removed from it (see Supplementary Material E2) and, as such, would not be expected to attenuate associations of speed with other variables.

Conversely to the results after age 11 IQ and education controls, the results after SES controls showed attenuations only of the relation of AL to speed and knowledge; the AL to *g* associations remained significant for 2 of the 3 models. However, even after attenuation, the AL-speed relation was still not 0 (range β values = −0.13 to 0.17). Again, these results may be because of a lack of statistical power to detect smaller effects. Nevertheless, the finding that the AL relation to speed and knowledge behaved differently than the relation to *g* after SES controls warrants further investigation.

Greater AL was associated with lower total brain and white-matter volumes, but not gray-matter volume, with the strongest effect seen on white-matter volume. Previous studies have found localized reductions in gray-matter volume as a result of accumulated adversity and stress (e.g., Ansell et al., 2012). We used only global measures of gray-matter volume that may be insensitive to localized effects. Similarly, whereas much of the research into AL, stress, and brain have implicated the hippocampus as a primary region of interest, and in the present study, the associations of AL with hippocampal volume in the left and right hemispheres were small (β = 0.09–0.14), dependent on the model being estimated and fluctuated between being significant or nonsignificant at an alpha level of 0.05. Again, the small effect sizes found in the present study may be a result of the use of a global measure of volume, masking more nuanced and specific associations that have been previously noted in the published literature (e.g., Apolstova et al., 2012).

Total brain and gray- and white-matter volumes were all significantly and positively associated with processing speed. Processing speed as a factor was defined by better scores on the speeded psychometric tests and quicker reaction times. As such, larger brain volumes were predictive of better speed performance. Importantly, this estimate of speed was independent of variance because of general cognitive ability. Interestingly, hippocampal volume in the left hemisphere had a significant negative association, suggesting that smaller volumes were associated with better speed performance. A similar pattern was observable with knowledge; however, the pattern of positive and negative associations was reversed. Larger left hippocampal volume was significantly associated with higher scores on knowledge, whereas smaller volumes for total brain and gray-matter volumes were associated with higher scores on knowledge. The independence of speed from general ability could, in principle, be part of the reason why a number of associations reported here differ from those reported elsewhere in the published literature.

However, a note of caution is warranted with respect to the findings of an AL association with processing speed and knowledge. The use of the bifactor model, although advantageous for decomposing variance because of general ability and specific abilities, requires a large number of cognitive tests to provide reliable estimates of the specific ability factors. The reliability of specific factor estimates can be assessed based on factor determinacies. Low determinacies suggest poorly measured constructs. In the present study, the determinacies for the specific ability factors were reasonable (range = 0.70–0.87), but not all were strong (usually >0.80 is considered to be good). Thus, although our results suggest some intriguing associations of processing speed and knowledge with AL and brain volumes, these results will require replication. However, the estimates remain valuable as few published studies correctly control for general ability in studying specific factors of cognitive functioning (see Booth et al., 2013a, 2013b).

Last, and importantly, inclusion of the volumetric measures of the brain did not fundamentally attenuate the associations between AL and cognitive ability. Therefore, despite AL being significantly associated with both current brain volumes and cognitive ability, the effects of AL on cognitive ability appear independent of brain volume.

The present study has a number of strengths. We used a large sample of generally healthy older adults with a wide array of measurements of cognitive ability, the brain, and AL. Collectively, this allowed us to model and test within a structural modeling framework a number of specific hypotheses concerning the associations between these constructs. Furthermore, as a birth cohort sample, we had a natural control for much of the effect of chronological age. As has been noted, research into AL in aging is limited and a primary area for development (Ganzel et al., 2010), as such the robust estimates provided in the present study offer significant contribution to the literature.

There are limitations of the present study. First, the mechanisms by which AL affects the brain is clearly finer grained than can reasonably be explored using gross measures of tissue volume. A large body of research in humans and animals has focussed on understanding these mechanisms, with recent results suggesting volumetric changes in gray-matter volume, for example, may be driven by loss of dendrites (Kassem et al., 2013). However, use of gross measure of volume can inform with respect to the potential negative health consequences of AL in later life. We used volumes of healthy tissue as variables of interest. However, within aging samples, it is important to note that volumes of healthy tissue are dependent on the volume of, for example, white-matter hyperintensities. That is, if tissue is hyperintense or has undergone atrophy, it cannot be healthy, and, therefore, our measures of healthy tissues may be proxies for the effects of AL on tissue integrity more generally.

In the present study, we used 10 biomarkers to assess AL. Whereas we consider the span of markers available to be suited to providing a good estimate of AL, we were not able to include markers of the sympathetic and parasympathetic nervous systems, known to be important in the stress response. Other biomarkers, including those involved in the hypothalamic-pituitary-adrenal axis, have also been included in the AL construct in some previous research (Seeman et al., 2001), and it would thus be of interest to test their associations with brain and cognitive measures in similar models to those tested here. Of particular interest would be the models including hippocampal volume because hippocampal structure has been found to be affected by presence of adrenal glucocorticoids (e.g., McEwen, 2001). In addition, our biomarker measures were imperfect; for instance, our participants were not fasting at the time of the lipid measures being taken, potentially influencing those measurements, particularly LDL and triglyceride, in unpredictable ways.

In addition, because there are sex differences in the subjective and objective experience of stress (e.g., Yang and Kozloski, 2011), future studies with large samples should examine whether sex moderates the size of the relations among AL, the brain, and cognitive abilities.

Our sample, although large and age homogenous, was generally healthy at approximately age 70 when recruited into the study. As such, it may be possible that the current sample does not include individuals who would be at the higher end of any measure of AL across the life course and thus may show the most severe effects of AL on both cognitive ability and brain volume measures. It is, therefore, possible that the estimates reported here are underestimates of the true effect size.

Whereas the data used in the present study are rich, and we were able to include childhood cognitive ability as a covariate in our models, we were limited with respect to the number of time points available and thus were unable to fully model the changes in AL (which is theorized to represent lifetime-accumulated stress but was only measured at 1 point), brain volume, and cognitive ability across multiple time points in old age. The inclusion of childhood cognitive ability provided a proxy for a full analysis of change; however, there are a number of methodological issues with assessing change both with only 2 time points and through using residual methods. However, even if an interpretation of our results with respect to change is considered problematic, the inclusion of prior ability as an important covariate and predictor of later life ability is an obvious strength of the present study. The LBC1936 is an on-going research study, and future investigations will extend the results reported here into such longitudinal designs. Finally, future research using large samples should add to the mediation models in the present study by also analyzing moderation, asking whether AL interacts with brain volume to affect cognitive outcomes. That is, it would be useful to test, in a large study with suitable power, whether associations with AL differ across the distribution of brain volume.

A number of the parameters reported in this article have been previously published by our group. Specifically, Booth et al. (2013b) reported measurement models of AL using the same marker set as used here. Booth et al. (2013a), (2013b) reported the bifactor measurement models for *g* that were used in the present study. Royle et al. (2013) reported the associations between total brain and white- and gray-matter volumes and *g* in males and females separately based on an overlapping sample from the LBC1936. Acknowledging this overlap with prior studies from our group, the current analysis focuses on the associations of AL as a multisystem summary of the accumulated effects of life stress, with both brain volumes and cognitive ability. The models presented test-specific hypotheses derived from the research literature in a sample drawn from an aging population, a heavily under-researched population in the AL literature (Ganzel et al., 2010).

The present study is the first to consider AL, cognitive ability, and neuroimaging measures of a range of brain volume measurements in a large, age-homogeneous sample of older adults. The results suggest that the cumulative wear and tear on the body from a lifetime of stress responsivity, AL, is associated with both brain structure and cognitive ability in later life but not with cognitive change from childhood to the early 1970s.

## Disclosure statement

None of the authors or their institutions have any actual or potential conflicts of interest to disclose.

## Figures and Tables

**Table 1:** Descriptive statistics of all study variables (for participants with MMSE scores >25)

Variables	*n*	Mean	SD	Skew	Kurtosis
Age (y)	633	72.49	0.72	0.01	−0.86
Brain imaging					
ICV (cm^3^)	633	1450.87	140.35	0.19	−0.33
Total brain volume (cm^3^)	630	1124.91	106.62	0.24	−0.03
White-matter volume (cm^3^)	628	496.79	83.07	0.45	0.48
Gray-matter volume (cm^3^)	629	500.15	71.10	0.18	0.76
Left hippocampal volume (cm^3^)	619	3.10	0.46	0.55	0.76
Right hippocampal volume (cm^3^)	619	3.33	0.45	0.35	0.54
Cognitive ability					
Logical Memory (immediate recall) WMS-III	633	46.26	10.08	−0.42	0.32
Logical Memory (delayed recall) WMS-III	633	29.25	7.86	−0.51	0.25
Verbal Paired Associates (first recall) WMS-III	625	2.84	2.31	0.61	−0.71
Verbal Paired Associates (second recall) WMS-III	622	6.43	2.08	−1.33	0.83
Spatial Span (forward) WMS-III	632	7.66	1.61	−0.07	−0.45
Spatial Span (backward) WMS-III	631	7.13	1.58	−0.01	−0.32
Verbal Fluency Total Score	632	43.60	12.42	0.29	0.12
National Adult Reading Test	632	34.91	7.75	−0.54	0.00
WTAR	632	41.54	6.45	−0.92	0.68
Simple Reaction Time Mean Score	633	0.27	0.05	1.72	4.59
Choice Reaction Time Mean Score	633	0.64	0.08	0.73	1.17
Inspection Time Total Correct Responses	621	111.66	11.31	−1.02	2.92
Digit Symbol WAIS-III^UK^	632	56.85	11.97	0.18	−0.25
Digit Span (backward) WAIS-III^UK^	633	7.96	2.26	0.31	−0.17
Block Design WAIS-III^UK^	631	34.57	9.96	0.45	0.08
Letter-Number Sequencing WAIS-III^UK^	633	11.14	2.91	0.43	0.35
Matrix Reasoning WAIS-III^UK^	632	13.57	4.86	−0.12	−0.94
Symbol Search WAIS-III^UK^	632	25.01	5.88	−0.26	0.83
AL biomarkers					
Fibrinogen	621	3.31	0.58	0.47	0.61
CRP	617	2.90	5.62	9.91	128.85
After log transformation	617	0.16	0.50	0.05	0.24
IL-6	617	2.05	1.80	3.05	12.49
After log transformation	617	0.20	0.29	0.36	1.26
BMI	633	27.80	4.38	0.89	2.24
Triglyceride	630	1.62	0.78	1.11	1.13
HDL	630	1.47	0.43	0.93	1.17
LDL	629	2.94	1.02	0.36	0.29
HbAlc	627	5.73	0.64	2.21	6.51
Mean DBP	631	77.44	9.68	0.20	0.00
Mean SBP	631	147.40	18.56	0.14	0.29
Medications		Yes	No		
Antihypertensive	633	332	301		
Anti-inflammatory	633	64	569		
Lipid lowering	633	214	419		
Insulin	633	8	625		
Other diabetes	633	40	593		
Any medications	633	559	74		
Demographics					
Years of education	633	10.84	1.14	0.71	2.31
Childhood SES	577	2.91	0.90	0.19	3.61
Adulthood SES	622	2.35	0.95	0.09	1.99
Sex		M	F		
	633	331	302		

Key: AL, allostatic load; BMI, body mass index; CRP, C-reactive protein; DBP, diastolic blood pressure; F, female; HbA1c, glycated hemoglobin; HDL, high-density lipoprotein; ICV, intracranial volume; IL-6, interleukin-6; LDL, low-density lipoprotein; M, male; MMSE, Mini-Mental State Examination; SBP, systolic blood pressure; SD, standard deviation; SES, socioeconomic status; WAIS, Wechsler Adult Intelligence Scale; WMS, Wechsler Memory Scale; WTAR, Wechsler Test of Adult Reading.

**Table 2:** Unadjusted Pearson correlations between primary constructs of interest based on pairwise complete observations (only including MMSE scores >25)

Variables	1	2	3	4	5	6	7	8	9	10	11	12	13	14
Age 11 IQ	—													
AL^a^	−0.11**	—												
General cognitive ability (*g*)^a^	0.65***	−0.12**	—											
Processing speed^a^	0.04	−0.11**	0.21***	—										
Verbal declarative memory^a^	0.03	0.04	0.14***	−0.17***	—									
Knowledge^a^	0.41***	−0.09*	0.22***	−0.30***	−0.02	—								
Nonverbal reasoning^a^	0.03	0.04	0.17***	0.08	−0.09*	−0.33***	—							
Total brain volume	0.08	−0.14***	0.23***	0.21***	0.05	−0.07	0.06	—						
White-matter volume	0.07	−0.24***	0.14***	0.11**	−0.04	0.06	−0.02	0.37***	—					
Gray-matter volume	−0.03	0.02	0.07	0.11**	0.12**	−0.10**	0.07	0.32***	−0.37***	—				
Hippocampal volume (L)	0.04	0.10*	0.01	−0.08*	0.10*	0.07	−0.02	0.24***	0.17***	−0.04	—			
Hippocampal volume (R)	0.03	0.09*	0.04	−0.03	0.05	0.04	−0.01	0.25***	0.21***	−0.01	0.74***	—		
Years of education	0.42***	−0.10**	0.45***	−0.05	0.04	0.42***	−0.04	−0.01	0.04	−0.06	−0.01	−0.02	—	
Childhood SES	0.17***	0.04	0.17***	0.00	−0.05	0.20***	−0.06	0.00	0.11*	−0.06	−0.08	−0.01	0.31***	—
Adulthood SES	0.34***	−0.09*	0.35***	0.02	0.00	0.25***	−0.02	0.05	0.11**	−0.03	0.06	0.07	0.41***	0.19***

***p < 0.001, **p < 0.01, and *p < 0.05. Key: AL, allostatic load; SD, standard deviation; SES, socioeconomic status; L, left; MMSE, Mini-Mental State Examination; R, right.

a Estimates of these variables were derived using regression-based factor scores from the measurement models estimated in Mplus.

## Appendix A Supplementary data

Supplementary Materials E1–E6
