# Supplementary material for: Association of allostatic load with brain structure and cognitive ability in later life
Source: Neurobiol Aging. 2015 Mar;36(3):1390–9. doi: 10.1016/j.neurobiolaging.2014.12.020 (PMC4353502; doi:10.1016/j.neurobiolaging.2014.12.020)
Supplement: Supplementary Materials E1–E6 [file mmc1.docx]

**Online Supplementary Material**

**Association of allostatic load with brain structure and cognitive ability in later life**

**Corresponding Author:** Tom Booth (tom.booth@ed.ac.uk). Centre for Cognitive Ageing and Cognitive Epidemiology, Department of Psychology, The University of Edinburgh, 7 George Square, Edinburgh, EH8 9JZ, UK.

**E1. Information on the extraction of allostatic load biomarkers**

Fibrinogen, triglyceride, HDL, LDL, and HbAlc were analysed at the Department of Laboratory Medicine, Western General Hospital Edinburgh. Fibrinogen assays were performed using an automated Clauss assay (TOPS coagulometer, Instrumentation Laboratory, Warrington, UK), with a coefficient of variability (CV) of 3.69%. Triglyceride, HDL and LDL were estimated using Abbott Architect c16000, with an assay CV of 3%. LDL was derived from total cholesterol, HDL and triglyceride (LDL=cholesterol– HDL–(triglyceride/2.19) mmol/L). HbA1c was assessing using Menarini HA8160 HBA1C analyser, with an assay CV of approximately 1% and a reference range of 4.0-6.0%. Note that participants were not fasting during these measurements.

CRP and IL6 were analysed at the University of Glasgow using high sensitivity ELISA from R&D Systems. For IL6, the minimum detectable dose (MDD) ranged from 0.016-0.110 pg/mL (mean=0.039 pg/mL). The intra-assay CV ranged from 6.9% to 7.8%, while the inter-assay CV ranged from 6.6% to 9.6%. For CRP, the MDD ranged from 0.005-0.022 ng/mL (mean=0.010 ng/mL). The intra-assay CV ranged from 3.8% to 8.3%, while the inter-assay CV ranged from 6.0% to 7.0%.

BMI was calculated as weight (kilograms) over height (metres squared) assessed during the physical examination. SBP and DBP were calculated as the average of three sitting readings taken using an Omron 705IT monitor.

**E2. Details of cognitive ability subtests**

Further details of the cognitive ability tests used in the current analyses are provided below.

1. *Logical Memory (WMS-III^UK^)* tests verbal declarative memory and provides immediate and delayed recall scores. Participants are asked to recall two 25 item stories which are read aloud by the examiner. Immediate (3 recalls total possible score 75) and delayed (2 recalls total possible score 50) scores were used.
2. *Verbal Paired Associates (WMS-III^UK^)* tests learning and memory. Participants are read lists of unrelated word pairs and are asked to recall the second of the pair when given the first. Immediate and delayed scores were used.
3. *Digit Span Backward (WMS-III^UK^)* tests working memory, with participants asked to recall increasingly long strings of numbers backwards.
4. *Spatial Span (WMS-III^UK^)* tests non-verbal/spatial learning and memory. The participant observes a series of blocks being touched and then has to touch the blocks in the correct order. The procedure is repeated with participants required to touch the blocks in reverse order. Forward and backwards recall scores were used.
5. *Block Design (WAIS-III^UK^)* participants are asked to use blocks to reproduce a diagram of a specific design.
6. *Matrix Reasoning (WAIS-III^UK^)* tests abstract reasoning. Participants view an incomplete pattern within a matrix, and are required to select from a number of options which piece completes the matrix.
7. *Digit Symbol Coding (WAIS-III^UK^)* tests speed of information processing. The participant is required to enter a symbol according to a particular number-symbol code. Participants are given 2 minutes to complete as many items as possible.
8. *Symbol Search (WAIS-III^UK^)* tests speed of information processing. Participants are given two target symbols and have to decide (yes or no) whether either symbol appears in a row of symbols. Participants are given 2 minutes to complete as many items as possible.
9. *Letter-Number Sequencing (WAIS-III^UK^)* is conducted by testers reading increasingly long lists of letters and numbers, with participants asked to recall the list immediately afterwards by stating the numbers in numerical order and then the letters in alphabetical order.
10. *NART and WTAR* are often used to estimate ‘prior cognitive ability level’ since they tap word recognition and pronunciation, a cognitive ability very robust against age- and trauma- related cognitive decline. Each requires the pronunciation of 50 irregular words.
11. *Verbal Fluency* tests executive function. Participants are asked to list as many words as they beginning with C, and then F, and then L, with 1 minute for each letter. Here we used a total score across letters.
12. *Reaction Time* tests speed of processing. Here we use both simple and 4-choice mean reaction time scores. In the simple task which had 20 trials, participants had to press a 0 key as quickly as possible when presented with a 0 on screen. In the 4-choice task, which had 40 trials, participants are presented with a 1, 2, 3 or 4 on screen, and have to press the corresponding button (labelled 1, 2, 3 and 4) as quickly as possible.
13. *Inspection Time* tests efficiency of visual discrimination. It is a forced-choice, two-alternative psychophysical task using the method of constant stimuli. In each of the 150 trials, participants are presented with two parallel vertical lines of very different lengths and, without time pressure, are asked to select which of the lines is longer. Stimulus durations range from 6 ms to 200 ms and stimuli were backward masked immediately after presentation.
14. *Moray House Test no.12* is a measure of general cognitive ability which contains items of a variety of different types including direction following, practical items, word-classifications, same-opposites, analogies, arithmetic, spatial skills, cypher decoding and mixed sentences. The MHT is often considered to be a test of verbal ability of reasoning.

**E3: Example of imaging**

**Figure 1:** Illustration of the segmentation of normal-appearing white matter (NAWM) and white matter hyperintensities (WMH) on a representative slice from two brain scans with medium WMH load. From left to right: MRI sequences to be fused, fused image in the red-green colour space and binary mask superimposed on the coloured image (WMH) and on the FLAIR image (NAWM).

**
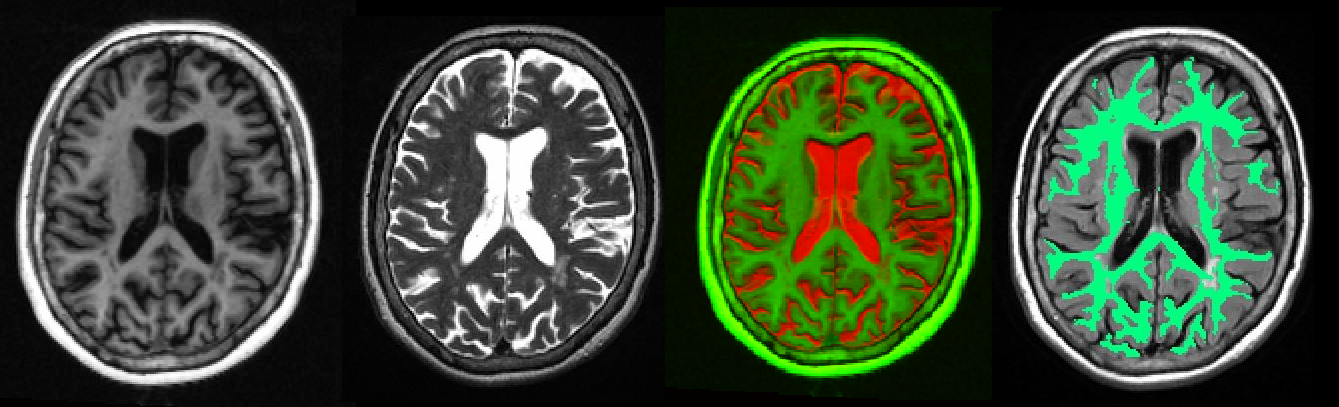
**

**
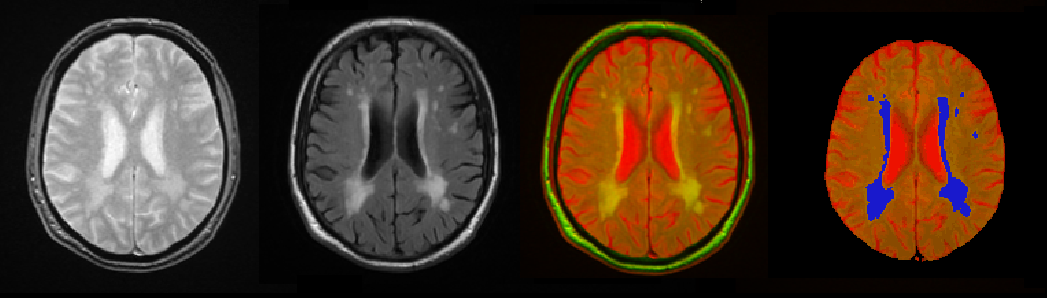
**

**E4. Description of multi-group SEM**

**Structural Equation Modelling: The Basics**

Structural equation modelling (SEM) combines factor analysis and path analyses to simultaneously estimate latent variables and the associations (both correlational and predictive) between them. Bollen (1989) provides the classical description of structural equation models with latent variables including discussion of the underpinning matrix algebra, model estimation, model identification and model fit.

Latent constructs are common in psychological literature where the substantive constructs of interest can often not be directly measured. As a result, the observed covariance’s (or correlations) between measured variables are hypothesized to be caused by a single underlying variable, the latent construct. A common example of a latent construct is general cognitive ability, or g. g is argued to be a common cause of the generally observed positive manifold of correlations between an individual’s scores on a battery of cognitive tests. Thus, when measured variables are hypothesized to have a common underlying cause, and they display moderate to high correlations, then measurement of a latent construct is plausible.

Supplementary Figure 1 - Supplementary Figure 1 shows a simple example of a SEM. Following convention, observed variables are denoted by rectangles, latent or unobserved variables by ellipses, directed or regression paths by single headed arrows and correlations by double-headed arrows.

Example of a SEM

X4

X6

X5

B

A

X1

X3

X2

A

A

A

A

A

C

X4

X6

X5

B

A

X1

X3

X2

A

A

A

A

A

C

In this model, latent variable (LV1) has three observed indicators X1 to X3, and latent variable (LV2) also has three indicators X4 to X6. Paths A are factor loadings, which range between -1.00 and 1.00 and represent the strength of the association between the measured variable and the latent factor. Factor loadings close to +/- 1.00 indicate strong associations.

A primary strength of structural equation modelling is that it takes account of error in measurement. When factor loadings do not equal 1.00, there remains a proportion of residual variance (sometimes defined as specific variance, sometimes error). In Supplementary figure 1, the residual variances of the measured variables X1 to X6 are denoted E1 to E6. Partialing out specific residual variance in this way means that the latent variables are error free representations of the theoretical common cause of the indicator variables.

Path B in Supplementary figure 1 shows a correlation (double headed arrow) between the residual variances of X1 and X2. Such paths represent shared variance between X1 and X2 which is not accounted for by the latent construct. For example, assume LV1 measures processing speed. X1 and X2 are experimental reaction time tasks, and X3 is a speeded paper pencil test. X1 and X2 will share some method variance based on the fact they are both experimental tasks. This shared association may need to be included in the model, and can be a priori, in order that the model fits the data suitably well.

Finally, path C in Supplementary figure 1 represents a regression path (single headed arrow) between the two latent variables. Here LV1 is regressed on LV2. If multiple variables were regressed on LV2, then the arrows and associated parameter estimates can be thought of as partial beta coefficients and interpreted accordingly.

SEM is a confirmatory method, that is, the inclusion or exclusion of a particular path in the model represents a hypothesis about the nature of its association with all other variables in the model (Bollen and Pearl, 2012). As such, SEM provides a framework for specifying and testing theoretical models and hypotheses between measured and latent constructs, and provides a framework for formal model comparison (see below).

Importantly, this note is intended as a basic description of the fundamentals needed to develop working knowledge of the models presented in the current manuscript, and is not intended as a technical discussion of SEM. For a detailed introduction to general latent variable models with structural equations see Bollen (1989) and for recent advances consult appropriate methodological journals.

**Multi-group Structural Equation Modelling (MG-SEM) and Tests of Parameter Equivalence**

MG-SEM is a methodology for testing the equivalence of structural models across groups. In the context of latent variables, MG-SEM is commonly referred to as measurement invariance. If measurement invariance is established across groups, then the latent constructs can be considered identical, and meaningful comparisons across groups, such as latent mean differences, can be made (French and Finch, 2006; Widaman 1993)

Invariance of latent constructs can be assessed at multiple levels, each providing a sequentially stricter test of invariant measurement (Widaman and Reise, 1997) Configual invariance requires the pattern of factor loadings to be the same across groups. Metric invariance requires the degree of the loadings to be equivalent across groups. Scalar invariance requires that the intercepts of the indicators are the same across groups.

Model fit indices are commonly used in order to assess whether the assumptions of invariance (constraints placed) have been violated. Cheung and Rensvold (2002) have suggested that changes of equal to or less than -0.01 in the CFI suggest that measurement invariance holds.

Once invariance has been established, equality constraints can be placed across groups in order to test whether a given parameter estimate is identical in each group. The plausibility of the equality constraint is assessed using a chi-square difference test. The imposition of a single equality constraint across groups results in a 1 degree of freedom change for the model (assuming 2 groups). Therefore, one can look at the difference in the chi-square statistic in these two groups and compare this difference to critical values for chi-square at 1 degree of freedom (the same logic holds if multiple constraints are placed at one time, e.g. one can look at critical values multiple degrees of freedom at a given level of significance).

**References**

Bollen, K.A., 1989. Structural equations with latent variables. Wiley, New York.

Bollen, K.A., Pearl, J., 2012. Eight myths about causality and structural equation models, in: Morgan, S. (Ed.), Handbook of Causal Analysis for Social Research, Springer, New York.

Cheung, G.W., Rensvold, R.B., 2002. Evaluating goodness-of-fit indexes for testing measurement invariance. Structural Equation Modeling. 9, 235-255.

French, B.F., Finch, W.H., 2006. Confirmatory factor analytic procedures for the determination of measurement invariance. Structural Equation Modeling. 13, 378-402.

Widaman, K.F., 1993. Common factor analysis versus principal component analysis: differential bias in representing model parameters? Multivariate. Behav. Res. 28, 263-311.

Widaman, K.F., Reise, S.P., 1997. Exploring the measurement invariance of psychological instruments: applications in the substance use domain, in: Bryant, K.J., Windle, M., West, S.G. (Eds.), The Science of Prevention: Methodological Advances from Alcohol and Substance Abuse Research, American Psychological Association Washington, DC.

**E5. Model fit tables for multi-group measurement structural models**

|  | χ^2^ | df | p-value | CFI | TLI | RMSEA | SRMR | AIC | BIC |
| --- | --- | --- | --- | --- | --- | --- | --- | --- | --- |
| *Cognitive Measurement Model* |  |  |  |  |  |  |  |  |  |
| M1: Multi-Group Configural | 470.252 | 228 | *p*<.05 | 0.956 | 0.941 | 0.057 | 0.046 | 28352.53 | 29025.91 |
| M2: Multi-Group Metric | 488.753 | 264 | *p*<.05 | 0.959 | 0.953 | 0.051 | 0.050 | 28315.91 | 28827.68 |
| *ΔM2 versus M1* | *18.501* | *36* | *p>.05* | *0.003* | *0.012* | *-0.006* | *0.004* | *-36.62* | *-198.23* |
|  |  |  |  |  |  |  |  |  |  |
| *AL Measurement Model^*^* |  |  |  |  |  |  |  |  |  |
| M3: Multi-Group Configural | 104.953 | 52 | *p*<.05 | 0.953 | 0.919 | 0.053 | 0.046 | 18980.46 | 19338.29 |
| M4: Multi-Group Metric | 118.555 | 62 | *p*<.05 | 0.950 | 0.928 | 0.050 | 0.058 | 18973.54 | 19285.49 |
| *ΔM4 versus M3* | 13.296^**^ | 10 | *p*=0.208 | -0.003 | 0.009 | -0.003 | 0.012 | -6.92 | -52.8 |

^*^As the number of participants with imaging data who were not taking medication was low (n=79), we could not estimate the allostatic load measurement model in this subset. As such, we estimated the stability of the bi-factor measurement model across medication status in the sample of 726 (On medication = 470; No medication = 256) participants reported on in Booth, Starr & Deary (2013).

^**^The AL measurement model was computed using robust maximum likelihood. As a result, the chi-square difference test is the Satorra-Bentler scaled chi-square difference test. This was calculated using freely available software (SBDIFF.EXE) available at <http://homepages.abdn.ac.uk/j.crawford/pages/dept/sbdiff.htm>

In the cognitive model, we constrained the pattern and magnitude of all factor loadings on both the general and specifics cognitive abilities. In the allostatic load model, as our interest was only in the general load variable, we constrained only the loadings on this factor and allowed the specific factors to vary by group.

**E6. Measurement Models for Cognitive Ability and Allostatic Load in the Whole Sample (n=633).**

*Allostatic Load*

Fibrinogen

CRP

IL6

Trig

HDL

LDL

HBa1c

BMI

0.63

0.77

0.53

0.62

-0.61

*0.16*

*0.12*

*0.16*

*0.10*

0.35

0.45

*-0.01*

0.34

-0.30

-0.37

0.39

0.57

DBP

0.99

SBP

0.54

*-0.08*

*Cognitive Ability*

NART

WTAR

Verbal Fluency

LM Immediate

LM Delayed

VPA Immediate

.71

.68

.12

.33

.37

.53

Digit Symbol

Simple RT

Choice RT

Symbol Search

Inspection Time

Matrix Reason.

Block Design

Digit Span Back.

Letter-Number

SS Forward

SS Backwards

.55

-.18

-.52

.46

.32

.16

.25

.11

.15

.53

.59

VPA Delayed

.72

.64

.39

.46

.62

-.23

-.39

.55

.64

.50

.48

.48

.59

.47

.60

.62

.36

.34

.31
